# Thyroid hormones according to gestational age in pregnant Spanish women

**DOI:** 10.1186/1756-0500-2-237

**Published:** 2009-11-26

**Authors:** Julia Pilar Bocos-Terraz, Silvia Izquierdo-Álvarez, Jose Luís Bancalero-Flores, Rosa Álvarez-Lahuerta, Ana Aznar-Sauca, Elisabet Real-López, Raquel Ibáñez-Marco, Virgilio Bocanegra-García, Gildardo Rivera-Sánchez

**Affiliations:** 1 Sección de Hormonas del Servicio de Bioquímica Clínica, Hospital Universitario Miguel Servet, Paseo Isabel la Católica 1-3, 50009 Zaragoza, Spain; 2 Departamento de Farmacia y Química Medicinal, Unidad Académica Multidisciplinaria Reynosa-Aztlán, Universidad Autónoma de Tamaulipas, C/16 y Lago de Chápala s/n, 88740 Reynosa, Mexico

## Abstract

**Background:**

Thyroid function changes during pregnancy and maternal thyroid dysfunction have been associated with adverse outcomes. Our aim was to evaluate thyroid hormones levels in pregnant women resident in Aragon, Spain.

**Findings:**

Samples for 1198 pregnant women with no apparent thyroid disorders were analyzed, using paramagnetic microparticle and chemiluminescent detection technologies, in order to determine levels of thyroid stimulating hormone (TSH), free triiodothyronine (FT3), free thyroxine (FT4), thyroid peroxidase antibodies (TPO-Ab), and thyroglobulin antibodies (Tg-Ab). Of the women in our sample, 85.22% had normal values for TPO-Ab and Tg-Ab and 14.77% had results revealing the presence of autoimmune diseases of the thyroid. The thyroid hormone reference values obtained according to gestational age (in brackets) were as follows: for free T3, values were 3.38 ± 0.52 pg/mL (<11 weeks), 3.45 ± 0.54 pg/mL (11-20 weeks), 3.32 ± 0.43 pg/mL (21-30 weeks), 3.21 ± 0.53 pg/mL (31-36 weeks), and 3.23 ± 0.41 pg/mL (>36 weeks); for free T4, values were 1.10 ± 0.14 ng/dL (<10 weeks), 1.04 ± 0.14 ng/dL (11-20 weeks), 0.93 ± 0.12 ng/dL (21-30 weeks), 0.90 ± 0.13 ng/dL (31-36 weeks), and 0.80 ± 0.21 ng/dL (>36 weeks); and for TSH, values were (μIU/mL): 1.12 ± 0.69 (<10 weeks), 1.05 ± 0.67 (11-20 weeks), 1.19 ± 0.60 (21-30 weeks), 1.38 ± 0.76 (31-36 weeks), and 1.46 ± 0.72 (>36 weeks).

**Conclusion:**

Pregnant women with normal antibody values according to gestational age had values for FT4 and TSH, but not for FT3, that differed to a statistically significant degree. The values we describe can be used as reference values for the Aragon region of Spain.

## Background

A dysfunctional thyroid gland during pregnancy can affect both mother and fetus [1-3]. One example is the maternal hypothyroidism and hyperthyroidism occurring at the beginning and end of pregnancy that may increase the risk of low birth weight and the likelihood of a Caesarean section [4,5]. For this reason, correct diagnosis is important so that any thyroid dysfunction can be treated. Nonetheless, the normal physiological changes that occur in pregnancy can make interpreting the test difficult [6], especially when there are no established reference levels for these hormones, as with the Aragon population.

Between 5% and 15% of pregnant women experience thyroid abnormalities, a fact which justifies screening by means of clinical laboratory testing [7,8]. In Spain, steady immigration flows in recent years have led to the development of a multiethnic population. Consequently, previous thyroid hormone reference values may be unreliable, since reference values tend to vary according to ethnic group [9-11].

The aim of this study was to evaluate 5 thyroid parameters for a normal pregnant population (median age, 31.47 years) resident in the Aragon region of Spain. The parameters measured were thyroid-stimulating hormone (TSH), free triiodothyronine (T3), free thyroxine (T4), thyroid peroxidase antibodies (TPO-Ab), and thyroglobulin antibodies (Tg-Ab).

## Methods

The study design and protocol were reviewed and approved by the independent ethics committee of Hospital Universitario Miguel Servet of Aragon, Spain, in accordance with the Declaration of Helsinki and the Nuremberg Code. Subjects in the study gave their informed consent.

Included in the study were 1198 pregnant women, with no apparent thyroid disorders, resident in the Aragon region of Spain and belonging to 5 self-reported ethnic groups, as follows: white; American-Indian; Arab; black; and Asian. The samples were analyzed using a high-performance immunoassay analyzer that uses paramagnetic microparticle and chemiluminescent detection technologies (ARCHITECT *i*2000_SR_, Abbott Diagnostics) [12]. The hormone samples were combined in the sample diluent with paramagnetic microparticles coated with anti-hormone antibodies to which the hormones bound. An acridinium-labeled anti-hormone conjugate was added following washing, then pre-trigger and trigger solutions were added to the reaction mixture. The resulting chemiluminescent reaction was measured in relative light units (RLUs). In our case, there was a directly proportional relationship between the amount of thyroid hormone present in the sample and the RLUs detected by the optical system.

The same method and procedure was used for the antibodies, with the only difference being that anti-human IgG acridinium-labeled conjugate was used instead of the anti-hormone conjugate.

The 5 analytes under consideration were determined in serum, with results expressed in units as follows (manufacturer reference limits in brackets): TSH in μUI/mL (4.94), free T3 in pg/mL (3.71), free T4 in ng/dL (1.48), TPO-Ab in IU/mL (<5.61), and Tg-Ab in IU/mL (<4.11).

Results for the 5 parameters (3 thyroid hormones and 2 thyroid antibodies) were expressed as mean (SD), median, 2.5th percentile, and 97.5th percentile for 5 gestational age intervals (less than 11 weeks, 11-20 weeks, 21-30 weeks, 31-36 weeks, and more than 36 weeks). Statistical analysis was performed using SPSS Version 13.0. The one-way ANOVA test was used to compare differences for the 5 gestational stages for a level of significance of *P *< .05.

## Results

Data for a total 1198 pregnant residents of Aragon (Spain) from a range of different ethnic backgrounds were analyzed. The sample population composition according to ethnic group was follows: white (85.16%); American-Indian (6.56%); Arab (3.61%); black (3.12%); and Asian (1.57%). Age range was 15 to 45 years, and the median was 31.47 years.

Thyroid antibody levels were normal in 1021 women, as follows: 330 women who were less than 11 weeks' pregnant; 200 women who were 11-20 weeks' pregnant; 208 women who were 21-30 weeks' pregnant; 269 women who were 31-36 weeks' pregnant; and 14 women who were more than 36 weeks' pregnant.

Table 1 shows the mean, standard deviation, median, 2.5th percentile, and 97.5th percentile for the 3 thyroid hormones and 2 thyroid antibodies according to gestational stage.

**Table 1:** Thyroid Hormone and Thyroid Antibody Values According to Gestational Age

Thyroid Hormones/Antibodies	Gestional Age, wk^a^	Mean	Median	2.5th %ile	97.5th %ile
**Thyroid-stimulating hormone (μUI/mL)**	<11	1.12 ± 0.69	1.00	0.10	2.65
	
	11 to 20	1.05 ± 0.67	0.92	0.03	2.57
	
	21 to 30	1.19 ± 0.60	1.12	0.12	2.64
	
	31 to 36	1.38 ± 0.76	1.29	0.23	3.56
	
	>36	1.46 ± 0.72	1.43	0.36	-

**Free triiodothyronine (pg/mL)**	<11	3.38 ± 0.52	3.37	2.34	4.34
	
	11 to 20	3.45 ± 0.54	3.49	2.24	4.43
	
	21 to 30	3.32 ± 0.43	3.33	2.47	4.18
	
	31 to 36	3.21 ± 0.53	3.24	2.25	4.16
	
	>36	3.23 ± 0.41	3.28	2.59	-

**Free thyroxine (ng/dL)**	<11	1.10 ± 0.14	1.10	0.83	1.38
	
	11 to 20	1.04 ± 0.14	1.02	0.77	1.34
	
	21 to 30	0.93 ± 0.12	0.93	0.70	1.14
	
	31 to 36	0.90 ± 0.13	0.89	0.66	1.17
	
	>36	0.80 ± 0.21	0.85	0.17	-
	
	<11	0.14 ± 0.21	0.09	0.00	0.68

**Thyroid peroxidase antibodies (UI/mL)**	11 to 20	0.17 ± 0.33	0.09	0.00	1.04
	
	21 to 30	0.15 ± 0.23	0.09	0.00	0.89
	
	31 to 36	0.19 ± 0.45	0.09	0.00	1.27
	
	>36	0.09 ± 0.08	0.08	0.00	-

**Thyroglobulin antibodies (UI/mL)**	<11	1.04 ± 0.65	0.82	0.41	3.11
	
	11 to 20	1.08 ± 0.70	0.86	0.34	3.31
	
	21 to 30	0.92 ± 0.55	0.75	0.37	2.60
	
	31 to 36	0.89 ± 0.52	0.76	0.38	2.45
	
	>36	0.90 ± 0.53	0.83	0.46	-

^a ^<11 weeks (n = 330); 11 to 20 weeks (n = 200); 21 to 30 weeks (n = 208); 31 to 36 weeks (n = 269); >36 weeks (n = 14).

**Figure 1:**
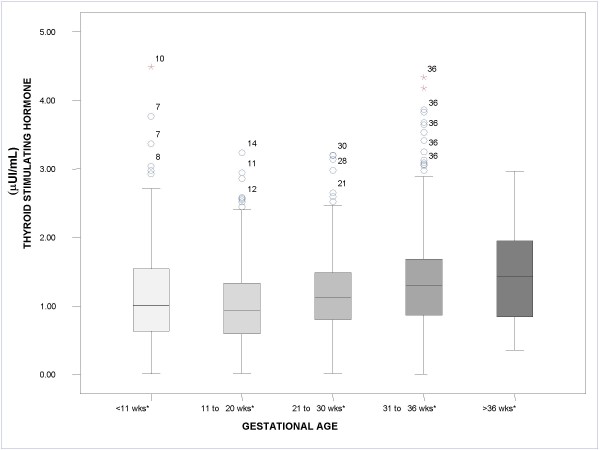
**Boxplot. Median value for thyroid-stimulating hormone according to gestational age**. *P < .05. The circles and stars represent distant values and outliers, respectively, and the adjacent numbers indicate the gestational week of the patient.

**Figure 2:**
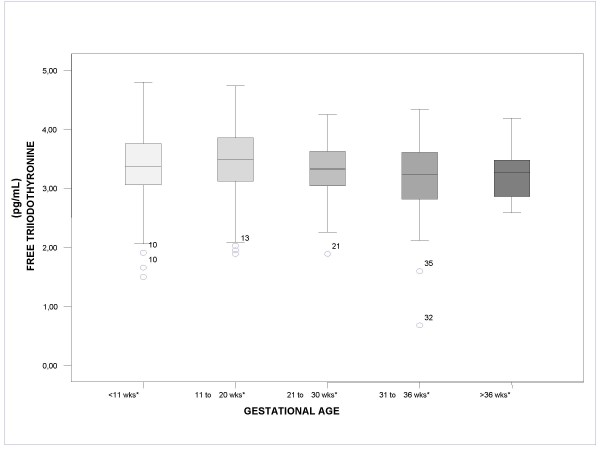
**Boxplot. Median value for free triiodothyronine according to gestational age**. *P < .05. The circles and stars represent distant values and outliers, respectively, and the adjacent numbers indicate the gestational week of the patient.

**Figure 3:**
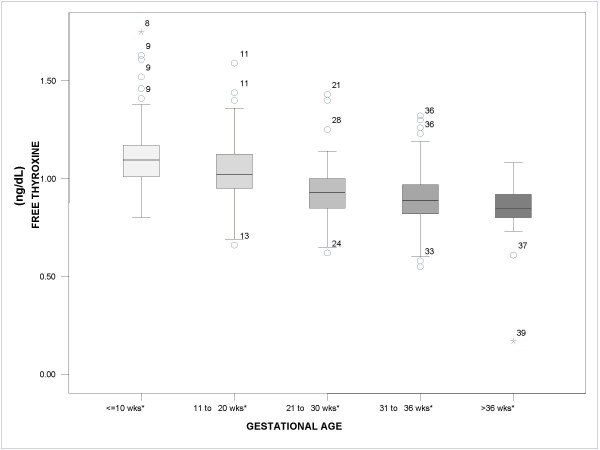
**Boxplot. Median value for free thyroxine according to gestational age**. *P < .05. The circles and stars represent distant values and outliers, respectively, and the adjacent numbers indicate the gestational week of the patient.

Figures 1, 2, 3 show the median values for each of the 5 gestational stages for TSH, free T3, and free T4, respectively. Figure 1 shows how median TSH values increased with gestational stage, with statistically significant differences for the gestational intervals defined (*P *< .05). In Figure 2, on the other hand, it can be observed that median free T3 values hardly changed from one interval to the next, with no statistically significant differences between the gestational intervals defined. Finally, Figure 3 shows how median free T4 values fell as gestational age advanced (*P *< .05).

It was observed that women in the population with antibody levels that were normal for the gestational stage had statistically significant different TSH and free T4 values (*P *< .001), but not free T3 values (*P *> .05). In relation to the age of the mother, statistically significant differences were only evident for TSH values (*P *< .05).

Table 2 shows the values obtained for the 5 parameters according to pregnancy trimester, with the ANOVA revealing significant differences between the 3 thyroid hormones for each trimester (*P *< .001).

**Table 2:** Thyroid Hormone and Thyroid Antibody Values According to Pregnancy Trimester

	Pregnancy											
	
Thyroid Hormones/Antibodies	First Trimester (<14 weeks) (n = 481)				Second Trimester (14 to 28 weeks) (n = 243)				Third Trimester (>28 weeks) (n = 297)			
	
	Mean	Median	2.5th %ile	97.5th %ile	Mean	Median	2.5th %ile	97.5th %ile	Mean	Median	2.5th %ile	97.5th %ile
**Thyroid-stimulating hormone** **(μUI/mL)**	1.08 ± 0.69	0.94	0.41	2.63	1.20 ± 0.62	1.13	0.15	2.59	1.39 ± 0.75	1.29	0.28	3.48

**Free triiodothyronine** **(pg/mL)**	3.41 ± 0.53	3.42	2.33	4.37	3.33 ± 0.45	3.34	2.42	4.20	3.22 ± 0.52	3.24	2.25	4.17

**Free thyroxine** **(ng/dL)**	1.08 ± 0.14	1.08	0.84	1.38	0.94 ± 0.12	0.94	0.70	1.16	0.90 ± 0.13	0.89	0.62	1.17

**Thyroid peroxidase antibodies** **(UI/mL)**	0.15 ± 0.26	0.08	0.00	0.74	0.16 ± 0.25	0.09	0.00	0.89	0.19 ± 0.43	0.09	0.00	1.20

**Thyroglobulin antibodies** **(UI/mL)**	1.05 ± 0.65	0.83	0.40	3.12	0.95 ± 0.62	0.76	0.34	2.65	0.89 ± 0.51	0.76	0.38	2.48

A total of 177 pregnant women had raised TPO-Ab and Tg-Ab values in gestational stages as follows: 63 women who were less than 11 weeks' pregnant; 33 women who were 11-20 weeks' pregnant; 34 women who were 21-30 weeks' pregnant; 45 women who were 31-36 weeks' pregnant; and 2 women who were more than 36 weeks' pregnant. Values for TSH, free T3, and free T4 for these women are given in Table 3.

**Table 3:** Thyroid Hormones Values According to Gestational Age in Pregnant Women With High Thyroid Antibody Values

Thyroid Hormone	Gestional Age, wk^a^	Mean	Median	2.5th %ile	97.5th %ile
**Thyroid-stimulating hormone (μUI/mL)**	<11	1.75 ± 3.64	1.25	0.1	13.5
	
	11 to 20	1.63 ± 1.30	1.44	0.0	-
	
	21 to 30	1.89 ± 0.96	1.62	0.4	-
	
	31 to 36	1.72 ± 0.91	1.72	0.2	4.5
	
	>36	1.19 ± 0.62	1.19	0.7	-

**Free triiodothyronine (pg/mL)**	<11	3.31 ± 0.58	3.36	1.7	4.4
	
	11 to 20	3.44 ± 0.65	3.59	1.7	-
	
	21 to 30	3.24 ± 0.51	3.15	2.3	-
	
	31 to 36	3.22 ± 0.47	3.24	2.4	4.3
	
	>36	3.02 ± 0.15	3.02	2.9	-

**Free thyroxine (ng/dL)**	<11	1.06 ± 0.15	1.06	0.8	1.4
	
	11 to 20	1.01 ± 0.15	1.03	0.6	-
	
	21 to 30	0.89 ± 0.97	0.87	0.7	-
	
	31 to 36	0.90 ± 0.13	0.86	0.7	1.3
	
	>36	0.90 ± 0.11	0.90	0.8	-

^a ^<11 weeks (n = 330); 11 to 20 weeks (n = 200); 21 to 30 weeks (n = 208); 31 to 36 weeks (n = 269); >36 weeks (n = 14).

Pregnant women with high TPO-Ab and Tg-Ab values had a mean TSH value that was higher than in pregnant women with normal TPO-Ab and Tg-Ab values, for all the gestational ages defined with 1 exception; this was the last interval (more than 36 weeks' pregnant), where the mean TSH value was lower. The free T3 mean value was lower in 4 gestation intervals (11-20 weeks, 21-30 weeks, less than 11 weeks, and more than 36 weeks) and slightly higher in 1 gestation interval (31-36 weeks). As for the mean free T4 value, this was slightly lower in all the intervals with the exception of the last gestation interval (more than 36 weeks), where it was slightly higher, and the second last gestation interval (31-36 weeks), where the mean value was the same as that for pregnant women with normal TPO-Ab and Tg-Ab levels.

In terms of ethnic origins, 92.7% of the women with high TPO-Ab and Tg-Ab values were white, 2.81% were Arab, 2.81% were American-Indian, 1.12% were black, and 0.56% were Asian.

## Conclusion

We measured TSH, free T3, free T4, TPO-Ab and Tg-Ab levels for a sample population resident in the Aragon region of Spain consisting of 1198 pregnant women representing different ethnic groups.

For the 1021 pregnant women with normal TPO-Ab and Tg-Ab levels, TSH and free T4 values were different depending on the pregnancy trimester, with statistically significant differences in these values for the 3 trimesters (*P *< .001), but not in free T3 values. It is important to have reference values for the 3 trimesters, particularly for TSH and free T4, and particularly for the first trimester, given that maternal thyroid hormone dysfunction may affect optimal development of the fetus [13,14].

Our results are different from those for other studies. Kurioka et al [15] reported significantly reduced levels of free T3 and free T4 and raised values of TSH as pregnancy progressed; and Kumar et al [16] reported TSH values that increased steadily with each trimester, and free T3 and free T4 values that increased in the first and second trimester but decreased in the last trimester. Our results are similar, however, to those reported by Marwaha et al [17], with the exception of the free T4 values, which, according to these authors, decreased as gestational age advanced. It was further observed from our data that only TSH showed significant differences (*P *< .005) according to the age of the mother.

Considering the full 9 months of pregnancy, and despite the fact that there was no evidence of the presence of thyroid antibodies or thyroid diseases, we observed 3 cases of high free T3 values (5.01 pg/mL, 6.09 pg/mL, and 7.1 pg/mL). For the mother with a free T3 value of 7.1 pg/mL, free T4 and TSH values were 2.13 ng/dL and 0.0018 μUI/mL, respectively. As for free T4, 2 first-trimester pregnant women with no evident pathologies had values of 1.63 ng/dL and 1.75 ng/dL, which, however, returned to normal levels in the second trimester.

Considering the entire gestational period, we observed 4 cases with free T4 levels above the manufacturer's upper limit (1.76 ng/dL, 1.75 ng/dL, 1.63 ng/dL, and 1.59 ng/dL), accompanied by TSH values below the lower limit. These may be cases of where physiological changes affected thyroid gland functioning and interpretation of the thyroid function tests [7,18,19].

Analyzing TSH data, the mean for our population was below the manufacturer's lower limit (<2.5 μUI/mL), and there were 5 cases with values above the upper limit (4.17 μUI/mL, 4.30 μUI/mL, 4.33 μUI/mL, 4.48 μUI/mL, and 5.17 μUI/mL); this percentage (0.59%) is lower than that reported for other studies [20].

The pathologies present in the women without thyroid antibody disorders were as follows (number of cases in brackets): hypothyroidism that resolved with treatment (6); hypothyroidism with gestational diabetes and low but normal TSH (1); thyroidectomy (1); goiter (2), multinodular goiter (1), cold nodule (1); and gestational diabetes (5).

We found that 177 (14.77%) of the pregnant women were positive for TPO-Ab and Tg-Ab, indicating a level of autoimmune diseases of the thyroid in our population that is very similar to that found in other studies [21-23].

The mean TSH in our population was found to be below the manufacturer's lower limit (<2.5 μUI/mL); nonetheless, similarly to findings in studies by other authors, we observed that high TPO-Ab and Tg-Ab values were associated with an increase in TSH values [24].

Finally, women with high levels of thyroid antibodies had medical histories as follows (number of cases in brackets): hypothyroidism (5, of which 2 had a previous history of miscarriage); thyroidectomy (2); simple goiter (1); chronic autoimmune thyroiditis (1); hyperthyroidism (1), thyroidectomy (2); fetal death (3); ectopic pregnancy (1); gestational diabetes (3); breast fibroadenomas (6), miscarriage (23); and repeated miscarriage (7).

In conclusion, of the 1198 pregnant women resident in the Aragon region of Spain analyzed in our study, 85.22% had normal TPO-Ab and Tg-Ab values and 14.77% tested positive for autoimmune diseases of the thyroid. In regard to the population with antibody values that were normal for the gestational stage, free T4 and TSH values, but not free T3 values, were statistically different.

The data from this study may be useful in establishing reference values for our population, in establishing limits in regard to detecting thyroid diseases such as Graves disease or Hashimoto thyroiditis, and in evaluating risk to the fetus or neonate as a result of maternal thyroid dysfunction.

## List of abbreviations

TSH: thyroid-stimulating hormone; T3: triiodothyronine; T4: thyroxine; TPO-Ab: thyroid peroxidase antibody; Tg-Ab: thyroglobulin antibody; RLU: relative light unit.

## Competing interests

The authors declare that they have no competing interests.

## Authors' contributions

**JPBT **carried out the assays and participated in designing the study. **SIÁ **carried out laboratory tests, participated in designing the study and performed the statistical analysis. **JLBF **participated in the sequence alignment and helped draft the manuscript. **RÁL **carried out the assays. **AAS **carried out the assays. **ERL **carried out the assays. **RIM **carried out the assays. **VBG **conceived the study, participated in its design and coordination and helped draft the manuscript. **GRS **helped draft the manuscript, revised it critically for intellectual content and gave final approval of the version to be published.

All authors read and approved the final manuscript.
